# Immunomodulatory, Antioxidant, Anticancer, and Pharmacokinetic Activity of Ulvan, a Seaweed-Derived Sulfated Polysaccharide: An Updated Comprehensive Review

**DOI:** 10.3390/md21050300

**Published:** 2023-05-16

**Authors:** Biswajita Pradhan, Prajna Paramita Bhuyan, Jang-Seu Ki

**Affiliations:** 1 Department of Biotechnology, Sangmyung University, Seoul 03016, Republic of Korea; pradhan.biswajita2014@gmail.com; 2 School of Biological Sciences, AIPH University, Bhubaneswar 752101, Odisha, India; 3 Department of Botany, Maharaja Sriram Chandra Bhanja Deo University, Baripada 757003, Odisha, India; prajnabhuyan2017@gmail.com

**Keywords:** ulvan, anticancer, cytotoxicity, antioxidant, immunomodulation, pharmacokinetics

## Abstract

Cancer is one of the most worldwide spread diseases and causes maximum death. Treatment of cancer depends on the host immune system and the type of drugs. The inefficiency of conventional cancer treatments as a result of drug resistance, nontargeted delivery, and chemotherapy-related negative side effects has caused bioactive phytochemicals to come into focus. As a result, recent years have seen an increase in research into screening and identifying natural compounds with anticancer properties. Recent studies on the isolation and use of polysaccharides derived from various marine algal species have revealed a variety of biological activities, including antioxidant and anticancer properties. Ulvan is a polysaccharide derived from various green seaweeds of the *Ulva* species in the family Ulvaceae. It has been demonstrated to have potent anticancer and anti-inflammatory properties through the modulation of antioxidants. It is vital to understand the mechanisms underlying the biotherapeutic activities of Ulvan in cancer and its role in immunomodulation. In this context, we reviewed the anticancer effects of ulvan based on its apoptotic effects and immunomodulatory activity. Additionally, we also focused on its pharmacokinetic studies in this review. Ulvan is the most conceivable candidate for use as a cancer therapeutic agent and could be used to boost immunity. Moreover, it may be established as an anticancer drug once its mechanisms of action are understood. Due to its high food and nutritive values, it can be used as a possible dietary supplement for cancer patients in the near future. This review may provide fresh perspectives on the potential novel role of ulvan, reveal a brand-new cancer-prevention strategy, and improve human health.

## 1. Introduction

Cancer is a major public health concern [1]. Off-target toxicity, drug resistance, and the financial burden of treatment costs pose potential obstacles in clinical oncology despite advancements in diagnosis, prognosis, and conventional therapeutic treatments [2]. In terms of global incidence and annual mortality, cancer has surpassed many other diseases and is now the second leading cause of death worldwide [3]. Global cancer statistics indicate that there were 9.6 million cancer-related deaths and an estimated 18.1 million cancer cases in 2018. Radiation therapy, immunotherapy, chemotherapy, and surgical methods have advanced to the clinical stage; however, despite extraordinary efforts over the past decades to improve conventional therapeutic approaches, some patients still lack treatment options [4,5]. The use of chemotherapy drugs at doses high enough to eradicate all drug-resistant subpopulations is constrained by side effects, including cardio-, hepato-, and neurotoxicity, along with nephron and life-threatening haematopoietic toxicity [6,7,8,9].

Over the past seven decades, natural compounds have been the primary source of innovative medication prospects [10,11]. Phytochemicals have emerged as prospective anticancer treatments, either alone or in combination with other chemotherapeutic drugs, due to their efficient tumour-targeting ability and low toxicity to normal tissues [10,11,12,13]. Phytochemicals act as chemopreventive and synergistic agents, increasing anticancer activity and decreasing chemotherapy-associated toxicity [14]. Pro-oxidative and antioxidative properties of phytochemicals positively regulate the homeostasis of reactive oxygen species (ROS), modifying apoptotic signals to prevent cancer [10,11]. In general, plant-derived polyphenols dynamically alter apoptotic and autophagic cell death signalling in cancer cells while blocking these signals in healthy organs surrounding the tumour to protect them [10,11]. Although few novel phytochemicals have been extensively studied in clinical settings, their potential to improve cancer therapy is promising [15].

**Figure 1:**
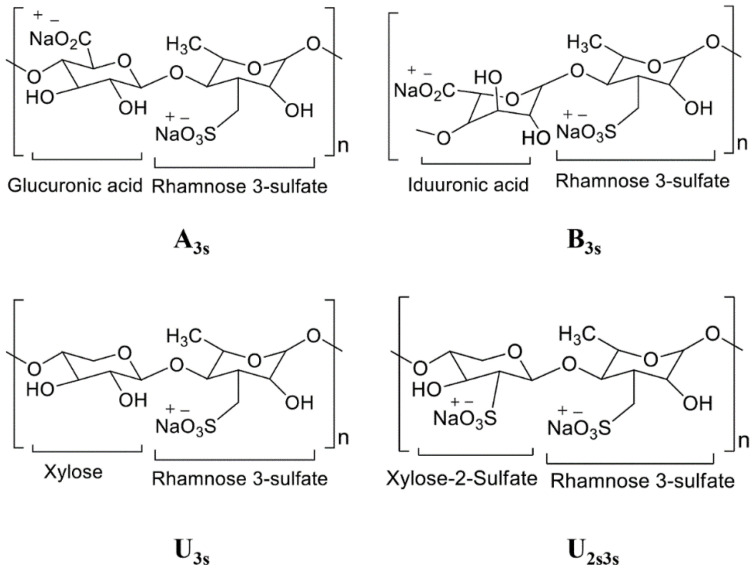
Structure of major repeating disaccharide units that comprise ulvan. The structure is drawn in Chemdraw and adopted and modified from [33]. Ulvanobiuronic acid A3s contains glucuronic acid attached to rhamnose 3-sulfate, while similar B3s also contain rhamnose 3-sulfate but have iduronic acid in place of glucuronic acid. Ulvanobioses are composed of rhamnose 3-sulfate attached to xylose. Xylose can contain a sulfate group, as seen in U2s, 3s.

Marine and freshwater ecosystems are rich in biodiversity and novel bioactive compounds [16,17,18,19,20,21,22,23,24,25,26,27,28,29,30,31]. Ulvan is a primary polysaccharide found in green seaweeds of the genus *Ulva* (family Ulvaceae). *Ulva* is a widely produced natural fiber and is considered an important food source. It also contains additional ingredients for biomass fuel production and therapeutic supplements [32]. Ulvan is a cell wall polysaccharide that makes up 9–36% of the dry-weight biomass of *Ulva* species and is mainly composed of uronic acids such as glucuronic acid, iduronic acid, sulfated rhamnose, and xylose [33]. *Ulva* species contain three other cell wall polysaccharides (cellulose, xyloglucan, and glucuronan) that make up to 45% of the dry-weight biomass when combined with ulvan [34]. Similar to ulvan, xyloglucan and glucuronan are soluble polysaccharides; however, they constitute a very small portion of cell wall polysaccharides [35]. Of the four polysaccharides found in the genus *Ulva*, only ulvan contains both rhamnose and iduronic acid in its cell wall [36]. The two primary repeating disaccharides are type A (A3S) and type B (B3S) ulvanobiuronic acid 3-sulfate [37,38,39]. Ulvan is a common food ingredient, and various studies have investigated its therapeutic potential [40,41]. Therapeutic applications of ulvan polysaccharides are gaining popularity in seaweed research [42]. The molecular structures of the chief repeating disaccharide units comprising ulvan are shown in Figure 1.

The increased use of artificial chemicals in cancer treatment has resulted in many side effects and risks. Therefore, there is a global tendency to return to natural resources that are therapeutically effective, socially acceptable, and economically accessible to those with a lower socioeconomic status. As a result, Mondal many researchers have focused on finding new anticarcinogenic compounds from algae and plants [43,44,45]. Algal-derived sulfated polysaccharides have been shown to function as free-radical scavengers and antioxidants in the prevention of oxidative damage in living organisms [46,47,48,49,50]. Therefore, we focused on ulvan as an anticancer agent and investigated its possible role in various cancers. Its antioxidant and immunomodulatory properties were also investigated. The study also focused on its food and nutritive values as a possible dietary supplement for cancer patients.

## 2. Methods

According to traditional Indian, Korean, and Chinese medicine, seaweeds are rich in bioactive molecules with diverse pharmacological activities. Using various search engines, we identified ulvan, a polysaccharide primarily derived from green seaweeds. We gathered information on ulvan and its potential immunomodulatory and anticancer activities from databases such as Google Scholar, PubMed, Web of Science, Science Direct, and Scopus. We used several keywords such as seaweed, ulva, ulvan, anticancer, antioxidant, immunomodulation, cytotoxicity, and pharmacokinetics. In this review, we chose only green seaweed-derived ulvan compounds with potential antioxidant, anti-inflammatory, and anticancer properties. The time frame we used for this review is from 1997 to 2023. Moreover, regarding the selection criteria, we focused particularly on ulvan with respect to cancer research. Additionally, we focused on cytotoxicity, immunomodulatory, antioxidant, pharmacokinetic, and apoptotic effects of ulvan.

## 3. Seaweeds: Potentially the Most Important Source of Bioactive Compounds

Lifestyle and dietary changes can prevent more than 33% of diseases such as cancer, diabetes, and chronic diseases linked with inflammation [51,52]. Nutritional supplements derived from natural sources may play important roles in disease prevention. Peptides, polysaccharides, amino acids, sterols, fatty acids, lipids, carbohydrates, polyphenols, vitamins, photosynthetic pigments, and minerals, found in marine algae, can act as potent antioxidants, and have antidiabetic and chemotherapeutic benefits in a variety of diseases [51,52].

### 3.1. Seaweeds as a Chief Source of Polysaccharides and Carbohydrates

Polysaccharides are abundant in seaweeds [53]. They make up about 4–76% of the total dry weight of the algae. Sulfuric acid polysaccharides, sulfated xylans, and galactans are examples of polysaccharides that are classified based on their chemical structures and are generally found in green algae. Brown algae also contain alginic acid, laminarin, fucoidan, and sargassan [54]. Red algae commonly contain agar, carrageenan, xylan, and floridean [55]. Due to the bioactive nature of these algal polysaccharides, they can be used as therapeutic candidates to address a wide range of human health issues [55]. For example, sulfated galactans such as carrageenans are widely used in the pharmaceutical and food industries. Brown seaweeds contain soluble fibers such as fucans, alginates, and laminarans, while red seaweeds contain soluble fibers such as sulfated galactans (carrageenans and agars), floridean starch, and xylans [56]. In addition to uronic acids, galactose, xylose, rhamnose, and arabinose, green algae also contain polysaccharides containing mannans, starches, xylans, and ionic sulfate groups.

Numerous polysaccharides are classified as dietary fibers and are divided into two categories: insoluble and soluble [57,58]. In contrast to their dry weight, seaweeds contain 25–75% dietary fiber, which is a higher percentage than that found in vegetables and fruits [59]. Algal dietary fibers have various health benefits, including antitumor, anticancer, anticoagulant, and antiviral properties [60]. Brown macroalgae contain numerous fucoidans in their cell walls [42,61]. Fucoidans have a wide range of biological effects, including antioxidant, anticancer, anti-inflammatory, antidiabetic, antiviral, antithrombotic, and anticoagulant properties [62,63,64,65]. They also influence the human immune system [62,63,64,65]. Furthermore, laminarin, which is abundant in brown algae and acts as an inhibitor of intestinal metabolism, is the second most abundant source of glucan [58].

### 3.2. Ulva and Its Food Value

Seaweed is increasingly being considered as a source of nutraceuticals and functional foods, where it can perform a variety of roles ranging from simple nutrition to sophisticated physiological mechanisms because it contains high levels of polysaccharides and natural fibers. In this context, the green seaweed *Ulva lactuca* has been widely used as a food and nutraceutical agent [66].

*Ulva* spp. are often rich in bioactive compounds known for their health-promoting properties and are traditionally used as a source of functional or nutraceutical foods. These products are sometimes consumed as whole foods or as dietary supplements. Seaweeds are assumed to contain several physiologically active compounds that can be employed as therapeutic agents in dietary supplements [67]. *Ulva* spp., such as *U. linza*, have evolved into supplements that can be used to treat a variety of ailments and as food and biomedical preservatives [68]. Numerous studies have established that *U. compressa*, *U. rigida*, and *U. intestinalis* can be employed as healing agents in antioxidant, anticancer, anti-inflammatory, antidiabetic, and antibacterial medicines [48,62,69,70,71,72].

## 4. Ulvan Has the Foremost Powerful Antioxidant Activity

In 2019 and 2020, several *Ulva* sp. sources were discovered to have antioxidant effects, including *U. rigida*, *U. australis*, *U. lactuca*, and *U. ohnoi* [73,74,75]. The antioxidant ability of ulvan was assessed using various in vitro methods, including DPPH (2,2-diphenyl-1-picrylhydrazyl), superoxide, hydroxyl, ferric reducing antioxidant power (FRAP), and lipid peroxidation inhibition experiments. Compared to other commonly used methods, such as reducing power and superoxide anion radical scavenging activity, the DPPH assay is the fastest approach for measuring antioxidant capabilities [74,76,77,78,79]. The antioxidant properties of ulvan from Ulva sp. have been associated with sulfate concentration and molecular weight [76,80,81,82]. Seaweeds such as *U. lactuca* can provide antioxidants. This alga exhibits antiradical properties by decreasing lipid peroxidation and enhancing antioxidant enzyme activity [83]. The degree of substitution of sulfate groups along the polymeric backbone is correlated with antioxidant activity [83].

Several studies have been conducted to compare methodologies and establish which method is more sensitive. As a method for tracking changes in peroxide generation, ORAC (Oxygen Radical Absorbance Capacity), FRAP, and β-carotene linoleic acid bleaching can be used [84,85]. The antioxidant effectiveness of ulvan has been compared with that of other substances such as BHA (Butylated hydroxyanisole), BHT (Butylated hydroxytoluene), and tocopherol. Although peroxide inhibition with ulvan (54.9%) was lower than that with BHA (73.20%) and BHT (69.40%), the differences were not statistically significant [77]. Ulvan exhibits antioxidative potential, as shown by a comparison of the numerous methodologies described above, according to an antioxidant testing study. To assess the antioxidant capabilities of ulvan, animal products, such as erythrocytes, and 2,2-azobis(2-amidinopropane) dihydrochloride (AAPH) tests have also been used [86,87]. Ulvan inhibits lipid peroxidation and lowers ROS formation by AAPH, as measured by thiobarbituric acid reactive substances (TBARS) in erythrocytes [86]. Sulfate and low-molecular-weight polysaccharides are used for antioxidant action [88,89,90]. The latter inhibits choline stresses and may be neuroprotective [38]. Malondialdehyde levels are reduced, whereas glutathione peroxidase (GSH), catalase (CAT), superoxide dismutase (SOD), telomerase, and other antioxidants are increased by oligosaccharide components [91,92]. Ulvan’s IC_50_ for radical activity is 623.58 µg/mL, whereas its IC_50_ for scavenging superoxide anions is 785.48 µg/mL. Pigments (chlorophyll and carotenoids), essential oils, and low-molecular-weight polysaccharides are the antioxidants found in *U. lactuca* [82].

The antioxidant properties of ulvan are also affected by the extraction process. Methanol extracts cause greater inhibition than water extracts, with a higher percent inhibition [93]. Furthermore, compared to acid extraction, enzymatic extraction results in a larger percentage of inhibition [94]. In addition to in vitro antioxidant studies, animals can be exposed to radicals such as thiacloprid and then treated with an extract [39,95]. Ulvan decreased oxidative stress in hypercholesterolemic mice by boosting the activity of antioxidant enzymes (110% for CAT, 77% for GPx, and 23% for SOD) and the levels of nonenzymatic antioxidants (GSH-stressed mice were treated with ulvan, which prevented abnormal lipid metabolism, controlled hepatic antioxidant defence mechanisms, and decreased lipid peroxidation) [96,97].

## 5. The Intricate Role of Ulvan as an Anticancer Agent

Cancer is a multistep process triggered by endogenous and external stimuli that frequently result in oxidative DNA damage and mutations that disrupt the usual regulatory pathways between cell differentiation, proliferation, and apoptosis [98]. Sulfated polysaccharides from green, brown, and red seaweeds, have sparked much interest in this context because of their anticancer properties [50]. In Swiss mice, a sulfated polysaccharide from *C. feldmannii* demonstrated anticancer efficacy in vitro and in vivo. This strengthens the immune system by increasing the production of OVA-specific antibodies (ovalbumin-specific antibodies) [99]. The anticancer properties of fucoidans have been demonstrated in several cancers, including lung, stomach, breast, and liver [62]. Fucoidans from Fucus vesiculosus demonstrate potent anticancer activity against HeLa G-63 and HepG2 cells. Fucoidan was also effective against HepG2 human liver cancer cells [100]. In this context, ulvan has received more attention than fucoidans and other sulfated polysaccharides.

The anticancer efficacy of ulvan has been studied in several ways. For example, ulvan can be used as a chemopreventive agent against liver cancer [74,101,102]. Ulvan contains sulfated polysaccharides that suppress hepatocellular carcinoma proliferation and induce apoptosis. The anticancer effect of ulvan has recently been discovered in *U. lactuca*, *U. australis*, *U. compressa*, *U. rigida*, and *U. ohnoi* [74,101,102]. HepG2 (hepatocellular carcinoma), MCF7 (human breast cancer), and HeLa (cervical cancer) are among the cell lines that have been tested [103]. Ulvan functions as an antiproliferative agent and causes apoptosis in malignant cells. Ulvan from *U. pertusa*, *U. lactuca*, *U. intestinalis*, *U. tubulosa*, *U. prolifera*, and *U. fasciata* [89,103,104,105,106,107,108,109] displayed anticancer activities in a range of cancer models, including murine sarcoma cancer cell line S180 [110], MCF7 (human breast cancer) [103,107], human cancer cell lines (e.g., HepG2 (hepatocellular carcinoma) [103,111,112], HeLa (cervical cancer) [103], MKN45 (human gastric cancer) [108], HCT-116 (human colon carcinoma), Caco-2 (human colon carcinoma) [111,113], AGS (human gastric carcinoma) [105,106], DLD1 (human colon carcinoma) [106,108], HT-29 (human colon carcinoma) [111,113], and some cancers in animal models (e.g., mice) [110]. However, human clinical trials have not been conducted to date. Ulvan, obtained from different sources, has varying degrees of anticancer activity. *U. lactuca* has been shown to be cytotoxic to several human cancer cell lines, including HepG2 (hepatocellular carcinoma), MCF7 (breast cancer), and HeLa (hepatocellular carcinoma) [103]. Ulvan reduced the in vitro viability of all three cancer cell lines to zero percent at a dose of 100 µg/mL. Similar investigations into the antitumor effects of ulvan in HepG2 and MCF-7 cell lines revealed increased expression of the proapoptotic tumour suppressor p53 and decreased expression of the antiapoptotic protein Bcl-2, supporting the theory that ulvan promotes apoptosis [113,114]. The anticancer activities of ulvan are listed in Table 1.

### 5.1. Anticancer Properties of Ulvan via Apoptosis

**Figure 2:**
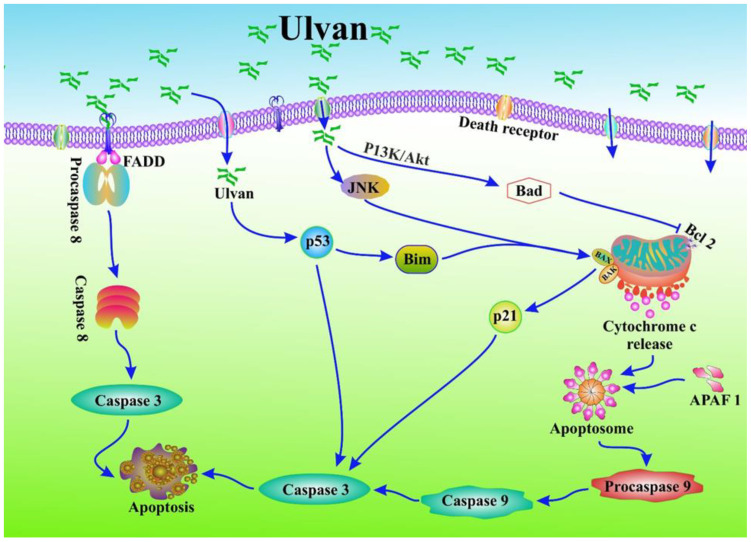
Ulvan modulates apoptosis to prevent cancer. Ulvan inhibits the expression of the antiapoptotic proteins Bcl-xl and Bcl-2 via intracellular oxidative stress, which in turn causes mitochondrial dysfunction. Like this, they increase Bax expression to promote apoptosis through cytochrome-C release, which causes the formation of an apoptosome, inducing procaspase-9 and caspase-9 and -3, resulting in apoptotic cell death. Moreover, it also induces procaspase-8 and caspase-8 and -3, and shows a caspase-dependent apoptotic cell death. Additionally, it triggers apoptosis via induction p21 and p53.

The tumour suppressor protein p53 and the antiapoptotic protein Bcl-2 are implicated in most human cancers [118]. p53 triggers an apoptotic process that prevents the growth of cells with damaged DNA or cancer cells by acting via either extrinsic or intrinsic apoptotic pathways involving p21 and Bax [119]. Bcl-2 functions primarily by inhibiting the apoptotic pathway [120]. The Bcl-2 gene product is a negative regulator of apoptosis that combines with Bax to counteract proapoptotic effects [121]. Bcl-2 is a crucial clinical prognostic marker for breast cancer [122,123]. Sulfated polysaccharides from algae increased p53 expression while decreasing Bcl-2 expression in mice with lung cancer [124]. The overall process by which ulvan modulates apoptosis for cancer prevention is shown in Figure 2.

Papillary cyst adenoma, hyperplasia of the ductal epithelial lining, intraluminal necrotic materials, and calcifications were observed in the breast tissues of the DMBA-administered group [114]. The DMBA-treated (2,4-Dimethoxybenzaldehyde) group that received ulvan polysaccharides did not develop these lesions [114]. DMBA-administered rats were treated with ulvan polysaccharides, which significantly increased the expression of the proapoptotic protein p53 and decreased the expression of the antiapoptotic marker Bcl-2 in the breast tissue, according to immunohistochemistry [114]. In the DMBA-administered control, ulvan polysaccharide treatment significantly decreased the elevated lipid peroxidation and suppressed antioxidant enzyme activity. Compared to the DMBA-administered control, DMBA-administered rats treated with ulvan polysaccharides had significantly decreased levels of the inflammatory cytokine’s tumour necrosis factor and nitric oxide. Ulvan polysaccharides may have chemopreventive effects during the initial and later stages of breast cancer. Increased apoptosis, decreased oxidative stress and inflammation, and a strengthened antioxidant system can exert these protective effects [114].

Ulvan polysaccharides reduce cell proliferation in *Ehrlich ascites* in a mouse model of Ehrlich Ascites Carcinoma (EAC). According to researchers, some EAC cells degenerate, while a large majority show phenotypic apoptotic signs, such as cell shrinkage, irregular shape, plasma membrane blebbing, cytoplasmic azurophilic lytic vesicles, apoptotic bodies, and fragmented nuclei [113]. In addition, an in vitro assay showed that *U. lactuca* polysaccharides significantly increased breast carcinoma cell lines’ (MCF-7) cytotoxicity and anticancer effects as concentrations increased from 25 to 200 µg/mL [114]. Recently, Ahmed and Ahmed found that *U. lactuca* polysaccharides (HCT116) were extremely toxic to EAC cells, hepatoma cell lines (HepG2), and colon carcinoma cell lines [113].

Ulvan from the seaweed *Ulva lactuca* has an antiproliferative effect on rat hepatocytes and lowers the levels of proliferating cell nuclear antigen (PCNA), suggesting that lower proliferation is accompanied by lower DNA replication [89]. Notably, in comparison with typical chemotherapy medications, numerous studies have found extremely low-to-moderate cytotoxic activity [105,109,110,125]. For instance, in human gastric carcinoma (AGS) and human colon cancer (DLD-1) cell lines, ulvan from *U. prolifera* showed only weak anticancer activity, inhibiting AGS cell growth by 10–26% at doses of 200–1000 µg/mL [106]. In addition, ulvan from *U. intestinalis* had no cytotoxic effects on sarcoma 180 tumour cells in vitro at concentrations of 50 to 800 g/mL but decreased tumour weight by 61 to 71% in mice when given at 100 to 400 mg/kg [110]. However, these findings do not rule out the possibility of using ulvan as an anticancer therapy.

When treated with ulvan, the thymus and spleen volumes increased, suggesting that the antitumor activity of this polysaccharide resulted from its immunomodulatory function. In conclusion, the anticancer effects of ulvan appear to be mediated by one or more of the following mechanisms: cancer cell death, decreased cancer cell proliferation, and stimulation of the innate immune response. Furthermore, the source and/or structure of ulvan affect the affected pathways [106,109,111,125].

According to preliminary findings, ulvan’s anticancer efficacy is influenced by both its molecular weight and degree of sulfation [106,109,111,125]. However, no definitive conclusions can be drawn regarding the effect of the structure of ulvan on its anticancer action. Owing to its broad-spectrum chemo preventative activities and low antiproliferative activity, ulvan is unlikely to replace known chemotherapy medications. However, its broad-spectrum chemopreventive activities mean that ulvan may be used as a combination therapy (e.g., antioxidant, anticancer, and immunomodulatory) [114,126]. Ulvan has intriguing prospective applications for cancer therapy. For example, pH-responsive polysaccharide nano systems suppress angiogenesis, and selenium-enriched polysaccharide–protein complexes are used for cancer treatment [127,128,129]. Ulvan bioavailability must first be established, and whether it has an additive effect on conventional chemotherapy drugs when used in combination therapies, before it can be used as a cotreatment or adjunct in anticancer therapies.

### 5.2. Cytotoxicity Activity of Ulvan: The Key to Anticancer Activity and Drug Discovery

Cancer is caused by the abnormal development of cells and tissues in the body. Cancer is caused by various endogenous and exogenous factors that frequently induce oxidative DNA damage, resulting in mutations that impair cell proliferation, differentiation, and death pathways [98]. Therefore, there is an urgent need to develop new drugs that are both economically and environmentally beneficial. Toxicity is a major concern during drug development. Ulvan has the potential to be used as a supplementary, therapeutic, and nutraceutical agent [98]. The cytotoxicity and therapeutic effects within this dose range (6.25–50 μg/mL) must be studied. In vitro with cells or in vivo with tested animals, cytotoxicity testing is performed [116,130].

A toxicity test must be performed and considered when establishing the stability and effectiveness of the product. Cytotoxicity tests were performed on cells in vitro and experimental animals in vivo [116,130]. Other toxicity experiments were performed using rat lung cells (L929), mammalian L6 cells [116,117,131], 3T3 fibroblasts, and HaCaT keratinocytes [86]. The most popular technique for toxicity testing is the MTT assay (3-(4,5-dimethylthiazol-2-yl)-2,5-diphenyltetrazolium bromide) [86,132]. Toxicity studies have used rat lung cells (L929), mammalian L6 cells, HaCaT keratinocytes, and 3T3 fibroblasts [116].

Before ulvan can be developed for biomedical applications, food, and supplements, it must have a low level of toxicity with no side effects. In a study on human L929 cells after 72 h of exposure, ulvan was metabolically active and showed no signs of reduced viability [116]. Several studies have examined ulvan’s anticancer activity in terms of toxicity and cell viability, specifically for anti-breast cancer, anti-colon, and anti-cervical cancer properties [103,115,133]. Ulvan has been tested in vitro on a variety of cancer cell lines, including HepG2, Caco-2 (human colon cancer), LS174 (human colon cancer), A549 (human lung carcinoma), Fem-x (malignant melanoma), K562 (chronic myelogenous leukaemia), HEp-2 (laryngeal epidermoid carcinoma), NCI-H292 (human colon cancer), and NCI-H292 (human colon cancer). It was also tested in rat cancer models such as diethylnitrosamine (DENA, 200 mg/kg intraperitoneally) and 7,12-dimethylbenz[a]anthracene (DMBA) [89,101,103,115].

Ulvan extraction from *Ulva* sp. was shown to be safe for mammalian L6 cells as a control, with no cytotoxicity (IC_50_ less than 90 mg/mL) even at the highest concentrations (10,000 mg/mL) in 3T3 cells [86]. Ulvan from *U. ohnoi* was tested on liver cells and found to be nontoxic [134]. Ulvan had weaker cytotoxic activity in cells A459 and LS174 (IC_50_ > 200 mg/mL), but it was more effective in preventing moderate cytotoxicity in Fem-x and K562 cells (IC_50_ 74.73 and 82.24 mg/mL, respectively) [82]. Ulvan was found to have an anticancer effect in MCF-7 and HCT-116 cells, with IC50 values ranging from 21 to 99 g/mL [115]. This appears to be due to the presence of sulfated polysaccharides with strong ligand interactions [88]. Moreover, a study concluded that the anticancer activity is also structural dependent and molecular weight dependent [103]. Ulvan was composed of rhamnose, galactose, xylose, manose, glucose (with a mole ratio of Rha: Gal: Xyl: Man: Glu equal to 1: 0.03: 0.07: 0.01: 0.06), uronic acid (21.5%), and sulfate content (18.9%) with a molecular weight of 347,000. This ulvan mainly consists of disaccharide [→4)-β-D-GlcA-(1→4)-α-L-Rha3S-(1→] and another minor disaccharide β-GlcA-(1→2)-α-Xyl and β-GlcA-(1→2)-α-Rha [103]. Ulvan suppressed hepatocellular carcinoma (IC_50_ 29.67 ± 2.87 µg/mL), human breast cancer (IC_50_ 25.09 ± 1.36 µg/mL), and cervical cancer (IC_50_ 36.33 ± 3.84 µg/mL). Ulvan has highly effective cytotoxic properties against hepatocellular carcinoma, human breast cancer, and cervical cancer [103]. The viability of HepG2, MCF7, and HeLa cells is directly related to the increase in ulvan concentration [103]. Human L929 cells are metabolically active and do not lose viability after 72 h of ulvan exposure [135].

Ulvan extracts have been shown to be safe for use as a control in mammalian L6 cells because they do not cause cytotoxicity (IC_50_ less than 90 mg/mL). Ulvan is not toxic to 3T3 cells at 10,000 mg/mL [86]. According to the results of a cytotoxicity test, ulvan from *U. lactuca* reduced cancer cell viability without affecting the viability of healthy cells [86]. Furthermore, low-molecular-weight polysaccharides (less than 5000 Da), typically oligosaccharides, inhibit Caco-2 cell proliferation [101]. Ulvan derived from albumin nanoparticles (NPs) has antiproliferative properties in MCF7 and HepG2 cells. Furthermore, they demonstrated the induction of apoptosis by increasing caspase-8 and caspase-9 levels [101]. Sulfated polysaccharides reduce oxidative stress and protect the liver from DNEA-induced damage [89]. They also improved the health of DMBA-treated (7,12-dimethylbenz[a]anthracene) mice by increasing apoptosis, decreasing oxidative stress and inflammation, and enhancing the antioxidant system [114].

## 6. Immunomodulating Activity of Ulvan

Humans use the immune system as a defence against invading agents. The modulation of the immune system is critical for disease management in humans. The importance of the immune system stems from the need to eliminate and control pathogenic and nonpathogenic microbes that can disrupt the body’s ability to maintain homeostasis [136]. For example, seaweed can be used to boost the immune system. *Ulva* sp. has immunomodulatory properties, and ulvan is its active constituent. Over the last five years, various Ulva species, most notably *Ulva intestinalis*, have been studied for their potential as immunomodulators. *U. intestinalis*, for instance, possesses both biochemical and immunomodulatory properties including in J774A macrophage cells where it increases the production of nitric oxide (NO) and of proinflammatory cytokines such as tumour necrosis factor (TNF-α) and interleukin-1β (IL-1β) [130]. Other studies support the in vitro findings that ulvan from *U. ohnoi* has immunomodulatory properties. To quantify ulvan’s in vitro immunomodulatory effect, the ability of the ulvan fraction to moderate inflammation produced by LPS-stimulated murine macrophages RAW264.7 was measured at the molecular level. All ulvan fractions showed no toxicity to RAW 264.7 cells at doses less than 100 g/mL for more than 48 h. The higher molecular weight ulvan fractions of interleukin-10 and prostaglandin E2 have anti-inflammatory properties at 100 g/mL [77]. Water-soluble sulfated polysaccharides were extracted from *U. intestinalis* and fractionated using a DEAE Sepharose rapid flow column to determine their molecular characteristics and macrophage cell-stimulating activity [137]. *U. ohnoi*’s immunomodulatory effects on Senegalese soles have also been studied in the fields of nutraceuticals and aquaculture (*Solea senegalensis*).) [134]. Furthermore, ulvan extracted from *U. ohnoi* to obtain fractions of various molecular weights (7, 9, 13, 21, and 209 kDa) demonstrated immunomodulatory activity [77]. Ulvan extracted from *U. ohnoi* displayed multiple immune system signalling pathways that were activated in different tissues as a result of intraperiotnean injection of ulvan into Senegalese sole juveniles, according to gene expression profiles [134]. Furthermore, ulvan modulates immune system pathways after challenge, and Phdp is a potential candidate nutraceutical and/or vaccine adjuvant for aquaculture [134]. In *S. senegalensis* macrophages, ulvan has a stimulatory effect that is enhanced when delivered via nanoparticles. Ulvan nanoparticles have the potential to act as macrophage activators and an immune stimulant in marine fish feed [138]. Dietary ulvan supplementation from U. clathrata increases the immune response in Nile tilapia [139]. The ulvan diet provides numerous health benefits against *F. columnare* by increasing antioxidant capacity, improving growth rate, innate–adaptive defence mechanisms, and modulating immune-antioxidant-related gene expression. Ulvan influences the innate–adaptive defence mechanism and expression of antioxidant genes in fish [140]. Supplementation with green macroalgae (Ulva intestinalis) increases the expression of immune-related genes such as lysozyme (Lyz) and interleukin 1 beta (IL-1β). Gutweed treatment significantly increased the expression of antioxidant-related genes (SOD and CAT) and growth hormone (GH) and insulin-like growth factor-I (IGF-1). Furthermore, dietary *U. intestinalis* improved immunity, and the same effects were observed on antioxidant and growth-related gene expression in zebrafish [141]. The Molecular weight of ulvan influences the inflammatory response of murine macrophages in vitro [142].

## 7. Pharmacokinetics: The Prevailing Study of Drug Discovery

Orally administered molecules with pharmacological activity or as nutrients must be released from the drug delivery system, absorbed from the gastrointestinal mucosal epithelium, delivered to the target cell or tissue after entering systemic circulation, and finally excreted from the body, either intact or in metabolite form. As they have β (1 β 4) connections, humans cannot digest ulvan polysaccharides from *U. lactuca*. Ulvan passes through the small intestine unmetabolized and is partially fermented by colon bacteria into short-chain fatty acids (SCFA) [143,144,145]. Ulvan has been shown to be beneficial to humans due to its immunostimulatory properties and ability to alter the human intestine microbiome. Ulvan is a soluble dietary fiber. Owing to their high inherent viscosity, aqueous media can slow digestion, reduce the bioavailability of minerals and other critical elements by chelation, and increase the quantity of Bifidobacterium and Lactobacillus in the caecum and large intestine, respectively. According to published data, ulvan is not destroyed in the human digestive system but is selectively absorbed in certain organs and tissues, with no obvious signs of harm to normal cells. The literature contains no data on absorption, distribution, metabolism, or excretion; however, cytotoxicity has been reported. Several active carbohydrate enzymes have been discovered that can hydrolyse or convert ulvan into oligomers [143,144,145]. More pharmacokinetics studies are required to conclude ulvan as an anticancer drug.

## 8. Conclusions and Future Perspectives

Ulvan, a polysaccharide derived from the green seaweed of the *Ulva* family, is a natural fiber with numerous health benefits. Ulvan has been studied in vitro and in vivo for its antioxidant, anti-inflammatory, antibacterial, anticancer, antiviral, and cytotoxic properties. Ulvan can be used as a polymer in pharmaceutical formulations to create smart films for bone-tissue engineering. However, the integrity of the ulvan structure must be preserved. Ulvan exhibited potent anticancer and immunomodulatory properties, among other biological functions.

Ulvan inhibits the abnormal proliferation of tumour cells while repairing cellular atypia and immune system damage caused by tumours. Owing to its potentially high medicinal value, ulvan merits further development as a biomaterial for human medical applications. However, its bioavailability must be investigated before it can be used for therapy. There is also a need to characterise the refining process. Several studies have investigated the biological activities and health benefits of ulvan. Therefore, elucidating the precise mechanism of action in animal models should be a priority in future studies. The distinct structure of ulvan meets the requirements of specific anticancer activities and processes for targeted applications. Furthermore, ulvan can be chemically modified to attach to functional groups and enhance its anticancer activity. Well-designed clinical trials are required to assess efficacy and safety in humans. Similarly, extensive clinical investigations of the pharmacokinetics, safety, and health benefits are required.

## Figures and Tables

**Table 1:** Ulvan anticancer activity.

Sl. No.	Source	Cell Lines	Test Type	Activity	IC _50_ µg/mL	References
1	*U. lactuca*	HepG2 cells	In Vitro	Cytotoxicity activity	29.67 ± 2.87	[103]
2	*U. lactuca*	Human breast cancer cells	In Vitro	Cytotoxicity activity	25.09 ± 1.36	[103]
3	*U. lactuca*	Cervical cancer cells	In Vitro	Cytotoxicity activity	36.33 ± 3.84	[103]
4	*U. lactuca*	MCF-7 cells	In Vitro	Anticancer activity with low IC_50_	21	[115]
5	*U. lactuca*	Colorectal HCT-116 cells	In Vitro	Anticancer activity with low IC_50_	99	[115]
6	*U. lactuca*	Noncancerous baby hamster kidney (BHK) cells	In Vitro	In Vitro cytotoxicity and proapoptotic activity	-	[101]
7	*U. lactuca*	Caco-2 cells (human colon cancer)	In Vitro	Anticancer activity with low IC_50_	-	[115]
8	*U. lactuca* and *E. intestinalis*	LS174 cells (human colon carcinoma)	In Vitro	Cytotoxicity activity	74.73 to 155.39	[82]
9	*Ulva lactuca* and *Enteromorpha intestinalis*	A549 cells (human lung carcinoma)	In Vitro	Cytotoxicity activity	74.73 to 155.39	[82]
10	*Ulva lactuca* and *Enteromorpha intestinalis*	Fem-x cells (malignant melanoma)	In Vitro	Cytotoxicity activity	74.73 to 155.39	[82]
11	*Ulva lactuca* and *Enteromorpha intestinalis*	K562 cells (chronic myelogenous leukaemia)	In Vitro	Cytotoxicity activity	74.73 to 155.39	[82]
12	*Ulva lactuca*	NCI-H292 cells (human lung mucoepidermoid carcinoma)	In Vitro	Cytotoxicity activity	-	[80]
13	*Ulva lactuca*	HeLa cells	In Vitro	Displayed cytotoxicity activity	-	[103]
14	*Ulva lactuca*	L929 cells (mouse lung)	In Vitro	Displayed in vitro cytotoxicity activity	-	[116]
15	*Ulva lactuca*	Mammalian L6 cells	In Vitro	Cytotoxicity activity	-	[117]
16	*Ulva lactuca*	HaCaT Keratinocytes	In Vitro	Cytotoxicity activity	-	[86]
17	*Ulva lactuca*	3T3 fibroblasts	In Vitro	Cytotoxicity activity	-	[86]

## Data Availability

Not applicable.

## Abbreviations

bFGF: basic fibroblast growth factor; BAX, Bcl-2-associated X protein; Bcl-2, B-cell lymphoma 2; Bcl-xl, B-cell lymphoma-extra-large; cAMP, cyclic adenosine monophosphate; EAC, Ehrlich ascites carcinoma; HIF-1, hypoxia inducible factor-1; IL2, interleukin-2; JNK, c-Jun N-terminal kinase; ROS, reactive oxygen species; TNF, tumour necrosis factor.
